# Lipid metabolism as a cell-state determinant in pulmonary fibrosis: from epithelial failure to ferroptosis-driven amplification

**DOI:** 10.3389/fphar.2026.1917792

**Published:** 2026-09-21

**Authors:** Er Lin, Zhehua Shao, Jiahao Liu, Zihan Yi, Lanying Shen, Yinghua Ying, Yue Hu

**Affiliations:** 1 Key Laboratory of Respiratory Disease of Zhejiang Province, Department of Respiratory and Critical Care Medicine, Second Affiliated Hospital of Zhejiang University School of Medicine, Hangzhou, Zhejiang, China; 2 Department of Geriatric Medicine, Second Affiliated Hospital of Zhejiang University School of Medicine, Hangzhou, Zhejiang, China

**Keywords:** alveolar epithelial cells, fatty-acid oxidation, ferroptosis, fibroblasts, lipid metabolism, lipid peroxidation, macrophages, pulmonary fibrosis

## Abstract

Despite 2 decades of mechanistic progress, pulmonary fibrosis still progresses in many patients, and established scar rarely resolves. Lipid metabolism has emerged as a unifying lens on this disease, but a useful synthesis requires recognizing that the relevant abnormalities are compartment-specific, stage-dependent, and unevenly supported by human evidence. Here we argue that lipid metabolism in pulmonary fibrosis is best understood as a determinant of cell-state stability, and that its failure plays out differently across three compartments. In alveolar epithelial cells, reduced lipogenesis, impaired fatty-acid oxidation, surfactant imbalance, and lipotoxic stress destabilize the regenerative AT2 state. This compartment exhibits the most consistent correlative human tissue associations reported to date. In fibroblasts, a shift away from fatty-acid oxidation toward anabolic and lipogenic programs likely stabilizes cells into a persistent myofibroblast identity at the expense of more reparative lipofibroblast-like states. In macrophages, altered lipid uptake, oxidized-lipid responses, and disturbed cholesterol handling support a profibrotic niche, with ontogeny and metabolic profile increasingly displacing classical M1/M2 categories. These compartment-specific programs converge on a shared downstream amplifier, namely phospholipid peroxidation and ferroptosis, where the strongest evidence again concerns injured epithelium. Therapeutic strategies group naturally into three tiers: clinical anchors with indirect metabolic relevance, including pirfenidone, nintedanib, and PDE4B inhibition; repurposed agents with metabolic rationale, including metformin, ezetimibe, and PPAR and LXR modulators; and mechanism-driven, largely preclinical strategies targeting mitochondrial-redox balance and ferroptosis. Reframing lipid metabolism as a cell-state determinant clarifies which abnormalities are actionable, in which compartments they matter, and when intervention is most likely to alter disease course.

## 1 Introduction

Pulmonary fibrosis is a common end-stage feature of several fibrotic interstitial lung diseases, including idiopathic pulmonary fibrosis (IPF) and non-IPF fibrotic ILDs, some of which later assume a progressive pulmonary fibrosis (PPF) phenotype (Liu et al., 2022; Wijsenbeek and Cottin, 2020; Raghu et al., 2022). As first-line antifibrotic therapeutics for interstitial lung disease, pirfenidone and nintedanib attenuate the rate of pulmonary function deterioration through combined antifibrotic and anti-inflammatory actions, but progression remains common and established scar seldom recedes. This unmet clinical gap has spurred sustained investigation into pathogenic pathways that mediate irreversible tissue remodelling during the pre-fibrotic stage (Wijsenbeek and Cottin, 2020; Raghu et al., 2022; Koudstaal et al., 2023).

One such mechanism is lipid metabolism. The lung depends on tightly regulated lipid programs for surfactant production, membrane turnover, energy supply, and regenerative repair, and disturbance in one of these domains often spills into the others (Koudstaal et al., 2023; Chen and Dai, 2023; Romero et al., 2018; Angeles-Lopez et al., 2025; Liang et al., 2024). What has become increasingly clear is that lipid metabolism in pulmonary fibrosis is not simply an energetic substrate of disease. It influences how individual cells maintain or lose their identity under chronic injury, and it shapes the boundary between successful repair and fibrotic progression.

Under physiological conditions, cells draw fatty acids from extracellular uptake and *de novo* synthesis, route them through elongation, desaturation, or β-oxidation, and balance these flows against storage and membrane assembly (Chen and Dai, 2023; Angeles-Lopez et al., 2025; Rodríguez et al., 2025; Paton and Ntambi, 2009; Houten and Wanders, 2010). This balance is also cell specific. Alveolar epithelial cells need a steady lipid supply to maintain surfactant homeostasis and to regenerate after injury, whereas stromal and immune cells use lipid pathways to maintain regular physiological function. Fibrosis therefore does not disrupt one shared lipid program. It disrupts several, and the pattern depends on cell type and local context (Koudstaal et al., 2023; Chen and Dai, 2023; Angeles-Lopez et al., 2025; Liang et al., 2024).

These disturbances are not distributed evenly. In the alveolar epithelium, reduced lipogenesis and defective fatty-acid oxidation (FAO) are tied to failed repair (Fu et al., 2023; Kim et al., 2021; Liang et al., 2023). In fibroblasts, metabolic rewiring helps stabilize the myofibroblast state at the expense of more reparative lipogenic identities (Sun et al., 2025; Jung et al., 2018). In macrophages, altered lipid uptake and cholesterol handling sustain a profibrotic niche, with cellular origin increasingly recognized as a stronger determinant than classical polarization labels (Ganeshan and Chawla, 2014; Braga et al., 2015; Misharin et al., 2017; Cheng P. et al., 2021). A recurring downstream consequence is lipid peroxidation. When iron handling shifts and antioxidant defenses falter, ferroptotic injury becomes more likely, particularly in stressed epithelium (Wang C. et al., 2023; Yao et al., 2025; Dixon et al., 2012). This offers a plausible bridge between metabolic dysfunction and structural progression.

Three propositions run through the discussion that follows. First, lipid metabolic remodeling in pulmonary fibrosis is compartment-specific and stage-dependent, and the most prominent human correlative signals are found in the alveolar epithelium. Second, phospholipid peroxidation and ferroptosis act as a shared downstream amplifier of compartment-specific metabolic failure rather than as an independent disease driver. Third, therapeutic strategies that engage this biology are best organized by their proximity to lipid metabolism and by the compartments and stages in which they are likely to matter.

## 2 Literature retrieval methods

PubMed/MEDLINE, Embase, Web of Science Core Collection and Cochrane Library were simultaneously retrieved, with the search completed on 15 July 2026. We covered literature published from January 2008 to December 2026; 2008 was selected as the starting point since landmark lipid-fibrosis studies and early ferroptosis-related iron-lipid oxidation phenotypes were first reported that year, whereas earlier papers lacked cell-specific mechanistic data relevant to our topic. Retrieval was divided into three theme-based stages combining standardized MeSH subject terms and supplementary free-text keywords linked by Boolean operators, the first set covering all forms of fibrotic interstitial lung disease including idiopathic and progressive pulmonary fibrosis, the second focusing on cell-type-specific lipid metabolic pathways such as lipogenesis, fatty acid oxidation, lipid peroxidation and cholesterol homeostasis across alveolar epithelial cells, fibroblasts and macrophages, and the third centering on ferroptosis regulatory molecules, antioxidant proteins and existing or candidate antifibrotic therapeutic agents. Eligible studies included cell/animal mechanistic experiments, human lung single-cell and lipidomic research, clinical cohorts, antifibrotic trials and high-quality reviews, and only English peer-reviewed full texts were retained. All included articles must establish cell-type-specific associations between pulmonary fibrosis and lipid metabolism, lipid peroxidation or ferroptosis, while pure lipid biochemistry without lung fibrosis model verification was ruled out. We also excluded conference abstracts, case reports, in vitro-only biochemical tests without lung tissue validation, duplicate articles, inaccessible full texts, and low-quality reviews without integrated mechanistic analysis. After initial database screening, all candidate records were independently assessed by one postgraduate student and one doctoral researcher through reading abstracts and core content to eliminate unqualified papers, and articles unanimously deemed eligible by both reviewers were aggregated into the final reference list.

## 3 Cell-type-specific lipid metabolic remodeling in pulmonary fibrosis

Lipid remodeling in pulmonary fibrosis is not a uniform process moving in parallel through every cell. It shifts with cell identity, local cues, and disease stage. The same metabolic change can therefore carry different meaning in epithelium, stroma, and immune cells, and it is increasingly clear that these changes do more than alter energy supply. They help determine whether each compartment retains a reparative cell state or drifts toward a persistent profibrotic one.

## 4 Alveolar epithelial cells

Alveolar epithelial cells, especially AT2 cells, sit at the center of pulmonary lipid homeostasis. They produce surfactant and serve as progenitor cells during alveolar repair, and both roles depend on intact lipid handling. In fibrosis, this program appears to break down early (Sunaga et al., 2013).

Reduced lipid synthesis emerges repeatedly in the epithelium. When lipogenesis falls, AT2 cells have fewer membrane lipids and fewer surfactant components available, and their capacity to absorb stress falls with it. Experimental work shows that restoring lipid synthesis can ease endoplasmic-reticulum stress and soften profibrotic responses (Romero et al., 2018; Liang et al., 2024). Specific lipid species also matter. Stearic acid attenuates profibrotic signaling in IPF-related epithelial models, suggesting that lipid composition, not just total lipid availability, shapes the epithelial response to chronic injury (Kim et al., 2021).

Fatty-acid oxidation is also impaired. Under normal conditions, FAO supports energy balance and helps epithelial cells enter a repair state. In fibrotic lungs, reduced FAO narrows that adaptive range. Recent work indicates that FAO is required for effective progenitor-cell repair, and that disrupted AMPK control of alveolar epithelial metabolism deepens this defect (Angeles-Lopez et al., 2025; Liang et al., 2023).

The epithelial phenotype is not defined by lipid deficiency alone. Defective lipid handling can also lead to intracellular lipid accumulation, with downstream lipotoxic stress, mitochondrial dysfunction, and greater susceptibility to oxidative injury. Lipid deficiency and lipid overload are therefore not necessarily contradictory observations. They likely mark different phases of the same failed repair process, with early-stage suppression of lipogenesis giving way to later FAO failure and lipid droplet accumulation in chronically injured AT2 cells (Kim et al., 2021; Sunaga et al., 2013).

The net consequence is a destabilization of the regenerative AT2 state. When lipogenesis falls, FAO is blunted, and lipotoxic stress accumulates, surfactant homeostasis becomes harder to maintain and the threshold for entering a successful repair program rises. Of the major pulmonary compartments, the alveolar epithelium remains the one in which lipid metabolic failure carries the clearest direct consequences and the most consistent correlative human observations.

### 4.1 Fibroblasts and myofibroblasts

Fibroblasts are the main effector cells of fibrotic remodeling, and their transition into myofibroblasts sits at the core of pulmonary fibrosis. This shift is not driven by signaling alone. It is accompanied by metabolic rewiring that helps consolidate the activated state (Ho et al., 2024; El Agha et al., 2017).

A recurring pattern is reduced fatty-acid oxidation alongside a shift toward glycolytic and anabolic programs. That combination supplies both energy and biosynthetic substrate for matrix production while helping lock in the myofibroblast identity (Sun et al., 2025). TGF-β is a major driver of this change. It promotes fatty-acid synthase-related programs and supports myofibroblast differentiation, while changes in AMPK-linked pathways reduce metabolic flexibility and make activated fibroblasts harder to reverse. Profibrotic signaling and metabolic change therefore reinforce one another (Jung et al., 2018; Ho et al., 2024).

Fibroblast lipid metabolism is not only about energy supply. It also bears on cell identity. Lipofibroblasts are more lipogenic and support epithelial repair, in part by providing lipid substrate to neighboring AT2 cells (O’Callaghan et al., 2024). Myofibroblasts, by contrast, are more matrix producing and more clearly pathogenic. Loss of the lipogenic stromal phenotype therefore favors fibrosis, whereas restoring it may help push fibroblasts away from a persistent profibrotic state (Bernard et al., 2015). Viewed in this way, fibroblast metabolic remodeling is best understood as a determinant of which stromal identity dominates rather than as a passive consequence of activation.

This is what keeps fibroblast metabolism therapeutically interesting. If fibrotic persistence depends in part on a stable metabolic state, altering that state may weaken myofibroblast survival or reopen more reparative stromal programs.

### 4.2 Macrophages

Macrophages shape the fibrotic microenvironment at several levels. They respond to epithelial injury, interact with fibroblasts, and influence how long inflammation and remodeling persist. In fibrosis, those functions are tightly tied to metabolism (Koudstaal et al., 2023; Chen and Dai, 2023).

One major source of heterogeneity is cellular origin. Tissue-resident alveolar macrophages support homeostasis in the uninjured lung. Fate-mapping experiments in mouse fibrosis models confirm that post-injury monocyte-derived alveolar macrophages stably engraft within lung tissue long-term, rather than acting as short-lived inflammatory intermediates, and form a core constituent of the profibrotic microenvironment (Braga et al., 2015; Misharin et al., 2017). Macrophage lipid trafficking networks undergo profound rewiring during fibrotic pathogenesis, as validated via lipidomic profiling of primary lung myeloid cells from IPF patients and fibrotic mice. Lipid uptake increases, responses to oxidized phospholipids become more prominent, and cholesterol trafficking and efflux are often impaired. In primary macrophage culture and bleomycin-injured lung tissue, CD36-related signaling captures this change well. Oxidized lipids can activate macrophages through scavenger-receptor pathways and drive the release of profibrotic mediators, so lipid accumulation is not simply collateral damage. It becomes part of the mechanism that helps keep remodeling in motion (Kwak et al., 2023; Parks et al., 2013). Together, these shifts favor a phenotype better suited to sustaining remodeling than to restoring homeostasis. The familiar M1/M2 distinction still has some descriptive value but is too narrow to capture the range of macrophage states seen in fibrotic lungs. Single-cell RNA-seq profiling of explanted IPF lung samples delineates discrete functional macrophage subpopulations, including SPP1-expressing profibrotic macrophages concentrated within fibroblastic foci, FABP4-positive resident homeostatic macrophages, and IFN signature-enriched inflammatory subsets, distinguished more by ontogeny and metabolic profile than by classical polarization labels (Jin et al., 2021; Ji et al., 2023; Morse et al., 2019; Adams et al., 2020). Different profibrotic programs also rely on different metabolic backbones. Some lean more heavily on fatty-acid metabolism and oxidative pathways, whereas others show stronger glycolytic features. The pattern depends on origin, local cues, and disease stage (Adams et al., 2020). Macrophages therefore do more than react to tissue damage. They translate altered lipid environments into persistent profibrotic signaling, although the causal weight of specific macrophage programs in human disease remains less certain than it is in the epithelium.

### 4.3 Crosstalk between epithelial, stromal, and immune lipid programs

Although individual-cell mechanistic descriptions are informative, they are insufficient to fully explain fibrotic disease. Compartment-specific lipid-mediated intercellular communication is now recognized as an important driver. Three central axes, namely the epithelium-macrophage axis, macrophage-fibroblast axis, and lipofibroblast-alveolar type II (AT2) niche axis, mediate cell-type-specific lipid metabolic perturbations and reinforce the profibrotic microenvironment. Bidirectional metabolic crosstalk along these axes escalates pathogenic lipid signals across epithelial, mesenchymal and immune compartments. We next dissect the regulatory mechanisms and experimental evidence for each axis. A structured summary of compartment-specific alterations and their evidence base is provided in Table 1, and an overview of cell-type-specific remodeling and intercellular crosstalk is shown in Figure 1.

**TABLE 1:** Cell-type-specific lipid metabolic alterations in pulmonary fibrosis and their functional consequences.

Cell type	Major lipid-related change	Functional relevance	Evidence base	Key translational limitation	Representative studies (key refs)
Alveolar epithelial cells (AT2)	• Reduced lipogenesis • Impaired fatty-acid oxidation (FAO) • Surfactant imbalance • Lipotoxic stress and lipid droplet accumulation	• Failed regenerative repair of the AT2 state • Increased susceptibility to oxidative injury and ferroptosis • Compromised surfactant homeostasis	Human tissue and single-cell data with experimental support; the most consistent correlative human signatures among the three compartments	Stage-specific mapping and intervention timing in human disease remain incomplete	(Romero et al., 2018; Angeles-Lopez et al., 2025; Liang et al., 2024; Rodríguez et al., 2025; Sunaga et al., 2013)
Fibroblasts and myofibroblasts	• Reduced FAO • Anabolic and glycolytic reprogramming • Loss of lipogenic stromal identity (lipofibroblast) • TGF-β-driven fatty-acid synthase activation	• Persistent myofibroblast differentiation • Sustained matrix production • Reduced metabolic flexibility, limiting reversibility	Predominantly mechanistic and preclinical, supported by stromal-lineage tracing	Direct human cell-state validation is less complete than for the epithelium	(Sun et al., 2025; Jung et al., 2018; El Agha et al., 2017; Bernard et al., 2015; Kheirollahi et al., 2019)
Macrophages	• Increased lipid uptake • Disturbed cholesterol trafficking and efflux • Heightened responses to oxidized phospholipids • Origin-specific metabolic programs (resident vs. monocyte-derived) • Emergence of SPP1+ profibrotic and FABP4+ resident-like macrophage subsets with distinct metabolic profiles	• Maintenance of a profibrotic immune niche • Altered mediator release shaping fibroblast activation • Persistence of monocyte-derived profibrotic states • Spatial enrichment of SPP1+ macrophages adjacent to fibrotic foci and abnormal epithelium	Human heterogeneity and single-cell data, with largely inferential causal assignment	Population heterogeneity and origin-specific effects complicate compartment-specific targeting	(Misharin et al., 2017; Sunaga et al., 2013; Kwak et al., 2023; Parks et al., 2013; Adams et al., 2020; Habermann et al., 2020)

Representative studies are selected as illustrative anchors rather than as an exhaustive citation list; reference numbers correspond to the main reference list. Evidence base summarizes the type of supporting data available for each compartment, distinguishing human tissue and single-cell evidence from primarily mechanistic or inferential support. AT2, alveolar type II, cells; FAO, fatty-acid oxidation; ER, endoplasmic reticulum; IPF, idiopathic pulmonary fibrosis; ECM, extracellular matrix; TGF-β, transforming growth factor β.

**FIGURE 1:**
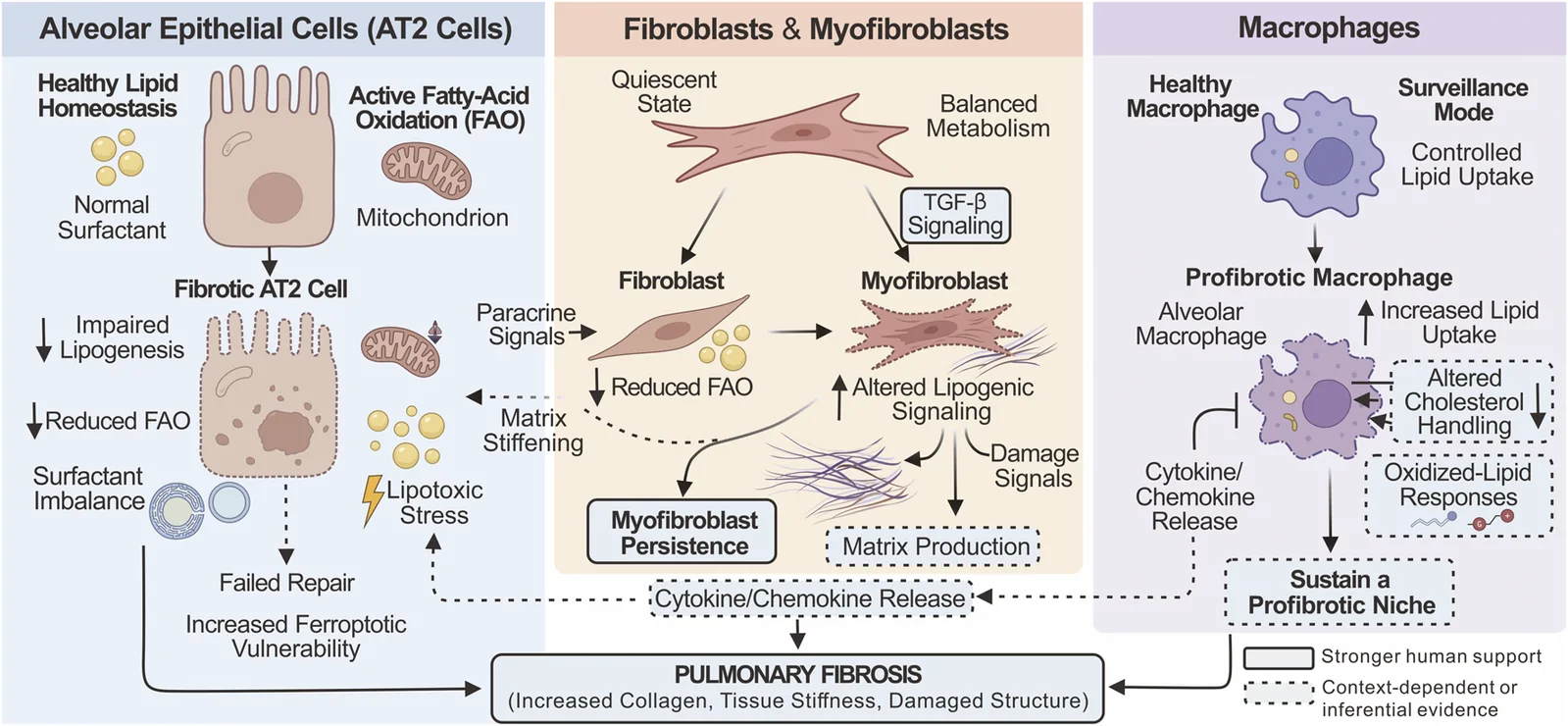
Cell-type-specific lipid metabolic remodeling and intercellular crosstalk in pulmonary fibrosis. Compartment-specific lipid abnormalities in alveolar epithelial cells (left), fibroblasts and myofibroblasts (middle), and macrophages (right), and the signals that link them. AT2 cells show impaired lipogenesis, reduced fatty-acid oxidation (FAO), surfactant imbalance, and lipotoxic stress, contributing to failed repair and ferroptotic vulnerability. TGF-β-driven activation of fibroblasts couples reduced FAO and altered lipogenic signaling to myofibroblast persistence and matrix production, with matrix stiffening signaling back to the epithelium. Profibrotic macrophages display increased lipid uptake, altered cholesterol handling, and oxidized-lipid responses, sustaining a profibrotic niche. These compartment-specific programs converge on the structural features of pulmonary fibrosis. Solid boxes denote findings with stronger human evidence; dashed boxes denote context-dependent or inferential evidence, reflecting unequal data maturity across compartments. FAO, fatty-acid oxidation; AT2, alveolar type II cells.

The first axis links injured epithelium to macrophages. Surfactant imbalance and lipid peroxidation in stressed AT2 cells release oxidized phospholipid species and altered surfactant components into the alveolar space. These species can engage scavenger-receptor pathways on alveolar macrophages, particularly CD36, and help drive a profibrotic mediator profile (Kwak et al., 2023; Parks et al., 2013). Single-cell and spatial studies of human IPF lungs identify SPP1+ profibrotic macrophages enriched adjacent to abnormal epithelium and fibrotic foci, consistent with epithelial-derived lipid signals shaping macrophage state *in situ* (Morse et al., 2019; Habermann et al., 2020).

The second axis runs between macrophages and fibroblasts. Lipid-laden and SPP1+ macrophages produce mediators that promote fibroblast recruitment, activation, and matrix production, and are often co-localized with CTHRC1+ or other profibrotic fibroblast populations at the periphery of fibrotic foci (Adams et al., 2020; Zuo et al., 2025). In this setting, macrophage lipid handling is not just a marker of altered immune state. It contributes to the mediators that keep the stromal compartment engaged.

The third axis is the lipofibroblast-AT2 niche. Lipogenic stromal cells supply lipid substrate that supports AT2 surfactant homeostasis and progenitor function, and loss of this lipogenic stromal support has been linked to impaired alveolar repair in aging and fibrotic lungs (Liang et al., 2024; Bernard et al., 2015). Failure of this niche therefore connects stromal metabolic identity directly to the epithelial repair failure described in Section 4.

These axes should be read as organizing models supported to different degrees rather than equally established mechanisms. Epithelium-macrophage and lipofibroblast-AT2 coupling are increasingly anchored in human single-cell and spatial data, whereas macrophage-fibroblast lipid signaling rests more heavily on inferential and animal-model evidence. A structured summary of compartment-specific alterations and their evidence base is provided in Table 1.

### 4.4 Lipid composition of bronchoalveolar lavage fluid (BALF) as a paracrine signalling reservoir

BALF functions as a shared lipid reservoir that enables metabolic crosstalk among alveolar epithelial cells, fibroblasts, and macrophages. Its lipid composition is dynamically shaped by surfactant secretion from AT2 cells, membrane lipid peroxidation during ferroptosis, and macrophage-mediated lipid turnover. Fibrosis remodels BALF lipid composition, reducing protective surfactant phospholipids while boosting oxidized polyunsaturated lipids (Sunaga et al., 2013). These bioactive lipids act as functional paracrine signals rather than passive injury markers to reshape cellular metabolism. For instance, oxidized phospholipids in BALF engage CD36 on alveolar macrophages to promote profibrotic polarization (Kwak et al., 2023; Parks et al., 2013), whereas excess sterol lipids impair epithelial fatty acid oxidation and reinforce lipogenic reprogramming in fibroblasts. Thus, BALF lipidomic profiling offers a minimally invasive window into compartment-specific metabolic derangements, and serves as a translational tool that bridges *in vivo* intercellular lipid signaling with the cell-type-specific pathways discussed above.

## 5 Lipid peroxidation and ferroptosis as a downstream amplifier of compartment-specific failure

Alveolar epithelial cells, fibroblasts, and macrophages do not share the same lipid dysfunction in fibrotic disease, yet their metabolic abnormalities converge on a common downstream problem: phospholipid peroxidation. In fibrotic lungs, changes in membrane lipid composition, iron handling, and antioxidant defense raise the likelihood that oxidative injury will spread through phospholipid-rich membranes (Cheng H. et al., 2021; Pei et al., 2022). Ferroptosis, an iron-dependent form of phospholipid peroxidation-driven cell death, provides a mechanistically coherent link between these metabolic disturbances and structural tissue injury (Song et al., 2025; Li Z. et al., 2024). It is best understood not as an independent disease driver, but as a downstream amplifier that converts compartment-specific metabolic failure into membrane damage, cell loss, and persistent profibrotic signaling (Kagan et al., 2017; Ursini et al., 2020; Wang B. et al., 2023). The major steps linking lipid peroxidation, impaired antioxidant defense, and ferroptosis in pulmonary fibrosis are summarized in Figure 2.

**FIGURE 2:**
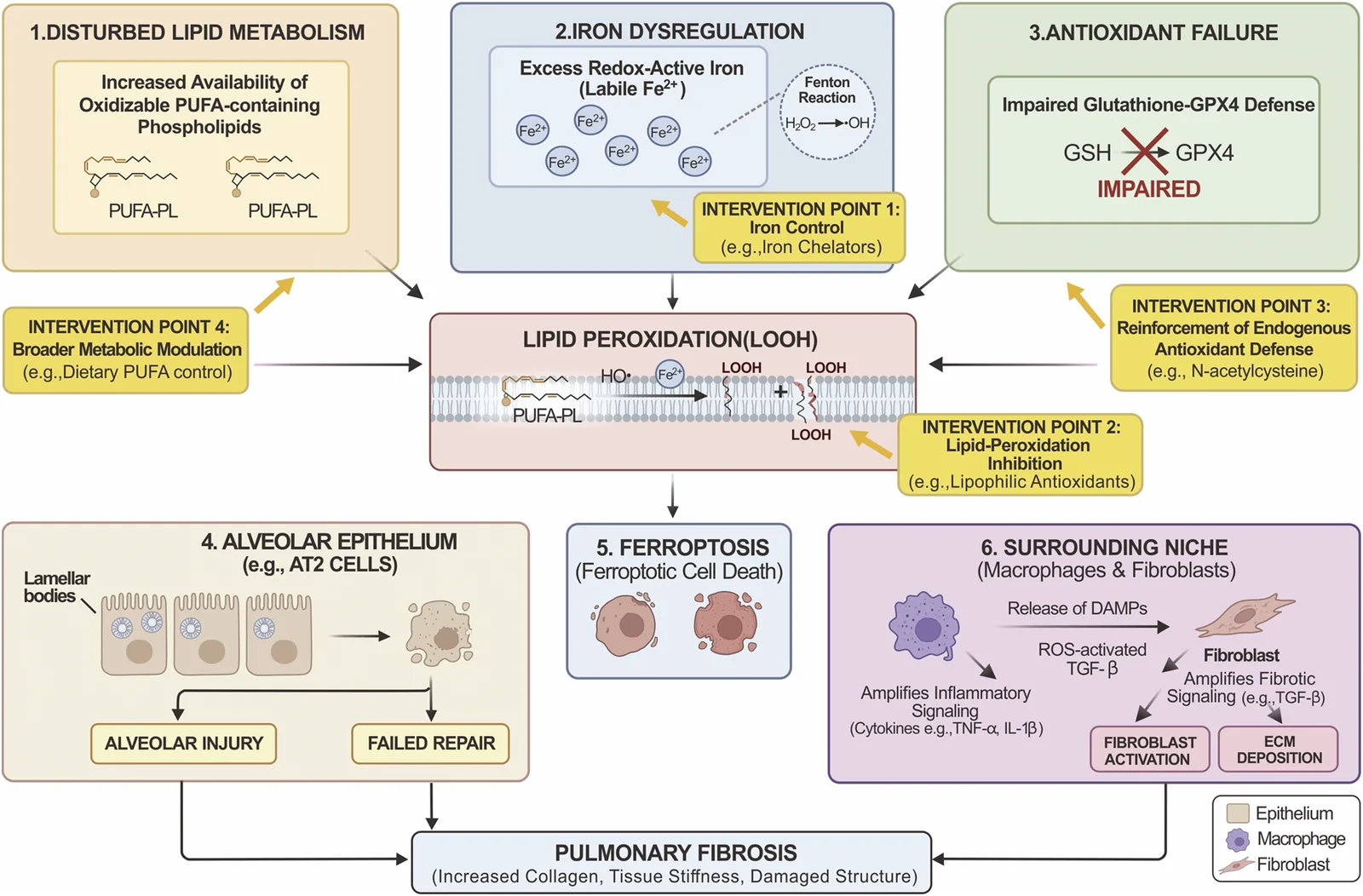
Lipid peroxidation and ferroptosis as a downstream amplifier of compartment-specific failure in pulmonary fibrosis. Three upstream programs converge on phospholipid peroxidation: disturbed lipid metabolism (orange) that increases oxidizable PUFA-containing phospholipid pools, iron dysregulation (blue) that expands the labile Fe^2+^ pool through impaired heme and ferritin turnover, and antioxidant failure (green) that compromises the glutathione-GPX4 axis and parallel FSP1/CoQ10 defense. The resulting lipid hydroperoxide (LOOH) accumulation drives ferroptotic cell death. In AT2 cells, ferroptosis contributes to alveolar injury and failed repair. Damage-associated molecular patterns and oxidized lipid species released by ferroptotic epithelium amplify inflammatory and profibrotic signaling in surrounding macrophages and fibroblasts, supporting extracellular matrix deposition. Four therapeutic intervention points are indicated (yellow): iron control, direct lipid-peroxidation inhibition, reinforcement of endogenous antioxidant defense, and broader upstream metabolic modulation. PUFA, polyunsaturated fatty acid; LOOH, lipid hydroperoxide; AT2, alveolar type II; GSH, glutathione; GPX4, glutathione peroxidase 4; FSP1, ferroptosis suppressor protein 1; ECM, extracellular matrix.

### 5.1 Molecular basis of lipid peroxidation and ferroptosis

Ferroptosis rests on three conditions: oxidizable membrane lipids, available redox-active iron, and insufficient antioxidant defense. When those factors converge, phospholipid peroxidation can spread beyond what the cell can contain.

Phospholipids containing polyunsaturated fatty acids are especially vulnerable. Once oxidation starts, chain reactions can propagate across membranes and disrupt both membrane integrity and signaling. Iron drives this process. Through redox chemistry, ferrous iron accelerates lipid peroxidation, so disordered iron handling leaves cells unusually vulnerable to oxidative damage. This is not a purely nonenzymatic event. Cellular enzymes, including acyl-CoA synthetase long-chain family member 4 (ACSL4) and lysophosphatidylcholine acyltransferase 3 (LPCAT3), determine how many oxidizable polyunsaturated lipid species are built into membranes and how readily they can be mobilized under stress (Song et al., 2025; Li Z. et al., 2024). Ferroptosis therefore reflects a specific imbalance rather than a generic surge in reactive oxygen species: vulnerable lipids are present, oxidative pressure rises, and the systems that normally contain phospholipid damage begin to fail (Wang B. et al., 2023; Li et al., 2023; Doll et al., 2019).

### 5.2 Antioxidant defense systems and their compartment-specific erosion

Sensitivity to ferroptosis depends not only on oxidative pressure but on whether protective systems remain intact. The glutathione-GPX4 axis is central; quantitative redox lipidomics and GPX4-knockout cell assays confirm its unique capacity to reduce toxic phospholipid hydroperoxides and block peroxidation chain propagation, a function sustained by cystine uptake, glutathione synthesis, and intracellular NADPH regeneration (Kagan et al., 2017; Ursini et al., 2020). When that metabolic support weakens, lipid-rich membranes become much easier to damage.

Parallel defense systems can compensate in some settings. CRISPR-mediated gene deletion in mammalian cell lines validates that the FSP1-CoQ10 axis operates independently of glutathione to suppress lipid-radical chain reactions (Li et al., 2023; Doll et al., 2019), while supplementary antioxidant modules including GCH1-BH4 and DHODH-driven mitochondrial CoQ reduction exert tissue-specific protective effects outside the lung. Their compartment-specific importance in pulmonary fibrosis remains largely uncharacterized, and to our knowledge no compartment-resolved data on FSP1 or DHODH function exist for human fibrotic lung disease.

In fibrotic lungs, chronic oxidative stress, mitochondrial dysfunction, and disturbed lipid handling can erode these safeguards. The result is not uniform vulnerability across the tissue, but compartment-specific susceptibility that likely matters most in repeatedly injured epithelium. The same logic may extend to macrophages and fibroblasts, but the supporting evidence there is less direct. From a therapeutic standpoint, that means preserving glutathione availability or reinforcing parallel redox defenses is unlikely to be useful in a blanket way. It will probably matter only in selected compartments and at selected stages.

### 5.3 Ferroptosis in pulmonary fibrosis across cellular compartments

Current evidence places ferroptosis at the interface between metabolic stress and structural tissue injury. The strongest support still concerns the alveolar epithelium. AT2 cells depend on stable lipid metabolism for surfactant homeostasis and repair, and they are highly sensitive to redox imbalance. When lipid peroxidation overwhelms local defenses, epithelial injury deepens and the threshold for failed regeneration drops (Ursini et al., 2020; Cheng et al., 2023). In bleomycin-induced lung fibrosis, interventions that limit ferroptosis or iron accumulation can lessen fibrotic change, strengthening the case that ferroptotic epithelial loss contributes to progression rather than merely reflecting tissue injury (Cheng H. et al., 2021; Pei et al., 2022).

Ferroptosis also shapes the fibrotic niche indirectly, and this is where it intersects with the crosstalk axes outlined in Section 4.3. Oxidized lipid species released by ferroptotic epithelium can engage scavenger-receptor signaling in alveolar macrophages, amplify inflammatory mediator release, and help sustain conditions that support fibroblast activation (Kwak et al., 2023; Parks et al., 2013; Pei et al., 2022). Ferroptosis therefore not only injures the epithelium directly. It feeds the epithelium-macrophage and macrophage-fibroblast signaling loops that keep remodeling in motion.

The case for ferroptosis in non-epithelial compartments still needs to be made carefully. Its contribution probably varies by cell type, disease stage, and model system, and support is strongest for epithelial injury. Whether comparable causal weight applies to stromal or immune compartments remains unsettled.

Read in this way, ferroptosis is best viewed as a downstream effector and amplifier of compartment-specific failure. It does not replace other disease mechanisms, but it helps explain how metabolic dysfunction is converted into persistent structural damage and how that damage feeds back into the profibrotic niche.

## 6 Therapeutic opportunities: direct and indirect targeting of lipid-centered fibrotic stress

Lipid metabolic remodeling is not only a descriptive feature of pulmonary fibrosis. It also opens a therapeutic frame, because altered lipid handling can shape epithelial repair, fibroblast activation, macrophage behavior, and ferroptotic susceptibility at the same time.

Current antifibrotic drugs slow disease progression but their effects remain partial. They do not reverse established fibrosis, nor do they directly correct the divergent metabolic abnormalities seen across epithelial, stromal, and immune compartments. That gap is part of why metabolism has become such a useful treatment lens. In epithelial cells, restoring lipid synthesis or FAO may improve repair; in fibroblasts, shifting metabolic state may weaken myofibroblast persistence; in macrophages, changing lipid uptake or cholesterol handling may help disrupt the profibrotic niche (Fu et al., 2023; Sun et al., 2025).

These approaches are not equally mature, and confusing biological plausibility with clinical proof has been one of the recurring problems of the field. We therefore group existing and emerging strategies into three tiers, defined by their proximity to lipid metabolism and by the strength of their clinical support. The first tier comprises clinical anchors with indirect metabolic relevance: established antifibrotics and PDE4B inhibition, where prospective trial data exist but the link to lipid metabolism is not the primary mechanism. The second tier comprises repurposed agents with explicit metabolic rationale, including metformin, ezetimibe, and modulators of PPAR and related lipid-sensing nuclear pathways, where the biological logic is direct but human efficacy is largely untested. The third tier comprises mechanism-driven, largely preclinical strategies that target the mitochondrial-redox network and ferroptosis. A summary of these tiers, their relationship to lipid biology, and the current stage of evidence is provided in Table 2.

**TABLE 2:** Therapeutic strategies targeting lipid metabolism, redox imbalance, and ferroptosis in pulmonary fibrosis.

Tier	Drug name	strategy/Mechanism of action	Current stage of evidence	Key limitation	Key references
Tier 1: Clinical anchors with indirect metabolic relevance	Pirfenidone/nintedanib	Inhibit fibrotic signaling and indirectly alleviate lipid-driven oxidative stress	Established clinical therapy	Do not directly correct divergent cell-specific lipid lesions	(Ishii et al., 2024; Hayek et al., 2024)
	PDE4B inhibition (e.g., nerandomilast)	Elevate intracellular cAMP to suppress myofibroblast activation and profibrotic inflammation	Prospective clinical support (phase III FVC benefit in IPF and PPF)	Clinical evidence strong, but lipid-metabolic link remains indirect	(Fu et al., 2015; Maher et al., 2025; Richeldi et al., 2025)
Tier 2: Repurposed agents with metabolic rationale	Metformin	Activate AMPK signalling to rescue mitochondrial function and restore cellular lipid homeostasis	Emerging, mostly translational/preclinical	Need clearer patient selection, timing, and human efficacy data	(Bernard et al., 2015; Rangarajan et al., 2018; Kheirollahi et al., 2019)
	Ezetimibe	Block sterol trafficking to lower lipotoxic injury and dampen lipid-provoked profibrotic responses	Translational/preclinical	Direct antifibrotic efficacy not established	(Seenak et al., 2022; Lee et al., 2024)
	PPAR agonists and lipid-sensing nuclear pathways (PPARγ, PPARα, LXRs)	Tune nuclear receptor activity to remodel compartment-specific lipid metabolic programmes	Mechanistic/preclinical	Strong biological rationale, marked context dependence	(Rodríguez et al., 2025; Houten and Wanders, 2010; Liang et al., 2023; Bernard et al., 2015)
Tier 3: Mechanism-driven, preclinical strategies	Mitochondrial/redox-modulating strategies	Improve mitochondrial integrity and mitigate harmful lipid peroxidation in fibrotic lung tissue	Mechanistic/preclinical	Often intersect with lipid biology without addressing a defined lipid lesion	(Koudstaal et al., 2023; Romero et al., 2018; El Agha et al., 2017; Tong et al., 2024)
	Ferroptosis-targeted strategies	Suppress iron-driven phospholipid peroxidation to defend alveolar cells against ferroptotic damage	Preclinical	Human therapeutic validation still lacking	(Wang B. et al., 2023; Nishizawa et al., 2023)
	Natural products/adjunctive interventions	Modulate multiple pathways to correct dysregulated lipid and redox homeostasis	Exploratory/preclinical	Mechanistic attribution and reproducibility often limited	(Xu et al., 2020; Shaker et al., 2026; Mei et al., 2025)

Strategies are grouped into three tiers defined by their proximity to lipid metabolism and the strength of clinical support. Relationship to lipid biology is rated as Direct (acts on a defined lipid-metabolic enzyme, transporter, or sensor), Intermediate (engages metabolic regulators that secondarily influence lipid handling), or Indirect (acts on signaling pathways with limited direct lipid-metabolic mechanism). FAO, fatty-acid oxidation; PPAR, peroxisome proliferator-activated receptor; AMPK, AMP-activated protein kinase; PDE4B, phosphodiesterase 4B.

### 6.1 Clinical anchors with indirect metabolic relevance: established antifibrotics and PDE4B inhibition

Pirfenidone and nintedanib remain the two main antifibrotic drugs used in fibrotic interstitial lung disease, especially IPF, with supportive data extending to broader progressive fibrotic ILD setting (El Agha et al., 2017; Pei et al., 2022; Cheng et al., 2023; Ishii et al., 2024; Hayek et al., 2024). Their major clinical contribution was to show that fibrotic progression can be slowed. Neither drug reverses established scar tissue. Neither is usually described as a metabolic therapy. Pirfenidone is discussed mainly in terms of anti-inflammatory and antifibrotic effects, and nintedanib is framed as a tyrosine kinase inhibitor acting on fibroblast-related signaling. Both, however, touch pathways that overlap with metabolism, oxidative stress, and biosynthetic activity.

Pirfenidone can reduce TGF-β-driven fibroblast activation, collagen production, and inflammatory signaling. In bleomycin mouse fibrosis models, oral pirfenidone alleviates AT2 iron overload, suppresses lipid peroxidation enzyme ACSL4 and upregulates antioxidant GPX4 to reduce ferroptotic epithelial damage (Pei et al., 2022). In NMDA-stimulated AT2 cell tests, pirfenidone decreases lipid peroxidation marker MDA and partially recovers GSH reserves (Cheng et al., 2023). Through those effects, it may also ease the conditions that favor persistent matrix production and oxidative injury, although direct evidence that pirfenidone restores lipid homeostasis is limited. Nintedanib blocks multiple growth factor receptor signals to restrain fibroblast proliferation and migration, indirectly reshaping the lung’s metabolic microenvironment. Because those pathways are tightly tied to anabolic remodeling and stress responses, nintedanib likely alters the broader metabolic environment of the fibrotic lung. Transcriptomic profiling of peripheral blood mononuclear cells from idiopathic pulmonary fibrosis patients receiving nintedanib supports drug-driven transcriptional shifts linked to profibrotic and metabolic pathways (Ishii et al., 2024). Still, such observations cannot substitute for lung-resident cell-level data to validate its direct modulatory effects on dysregulated pulmonary lipid metabolism (Ishii et al., 2024; Hayek et al., 2024).

PDE4B inhibition is a more recent addition to this tier. By blocking cyclic nucleotide breakdown, PDE inhibition changes intracellular signaling, especially through cyclic AMP, and affects cytokine production, proliferation, and tissue remodeling across epithelial, immune, and mesenchymal compartments (El Agha et al., 2017). Among PDE family members, PDE4B has drawn the most attention because of its relevance to immune and stromal cell behavior. The clearest recent evidence comes from nerandomilast, where phase III trials in idiopathic pulmonary fibrosis and progressive pulmonary fibrosis showed a smaller decline in forced vital capacity over 52 weeks than placebo (Maher et al., 2025; Richeldi et al., 2025). Among the strategies discussed in this review, PDE4B inhibition now has some of the strongest prospective clinical support beyond standard antifibrotic therapy, even though its mechanistic link to lipid metabolism remains indirect. A more cautious reading is that PDE4B inhibition reshapes the signaling environment in which metabolic injury is amplified rather than correcting lipid metabolic defects.

The limitations of this tier are equally important. Pirfenidone, nintedanib, and PDE4B inhibition do not fully restore epithelial repair, reverse stromal activation across compartments, or selectively correct the divergent metabolic programs seen in different cell types. Those gaps help explain the continuing interest in more targeted metabolic strategies and, eventually, in combinations that pair clinical anchors with direct metabolic interventions.

### 6.2 Repurposed agents with metabolic rationale: metformin, ezetimibe, and lipid-sensing nuclear pathways

Some of the more interesting metabolic ideas in pulmonary fibrosis come from drug repurposing. These agents share a feature: each has an explicit metabolic mechanism, each is familiar in clinical medicine, and each has biologically plausible but clinically unproven relevance to fibrosis.

Metformin has received the most attention. Its effects extend well beyond glucose lowering and include AMPK-related signaling, mitochondrial function, and redox balance. In experimental lung fibrosis, metformin has been reported to shift stromal cells away from profibrotic myofibroblast programs and toward a more lipogenic state, and it is often discussed as a possible pro-resolution strategy (Rodríguez et al., 2025; Rangarajan et al., 2018; Kheirollahi et al., 2019). The main limitation is that most of this evidence remains preclinical. How well these effects translate to human fibrotic lung disease is still uncertain, and response will probably depend on dose, timing, and disease stage.

Ezetimibe represents a different kind of metabolic intervention. It acts through cholesterol handling rather than AMPK signaling. That matters because fibrosis is not only about fatty-acid metabolism. Cholesterol trafficking and sterol balance may also shape epithelial stress, inflammatory tone, and profibrotic signaling (Seenak et al., 2022). Recent translational and preclinical work suggests that ezetimibe may be relevant to idiopathic pulmonary fibrosis (Lee et al., 2024). This mechanistic rationale draws on its ability to modulate intestinal cholesterol absorption and systemic sterol homeostasis, which could in turn mitigate cholesterol-driven epithelial injury and profibrotic inflammatory cascades within the lung. Nevertheless, the field is still early, and direct clinical antifibrotic efficacy has not been established.

The PPAR family sits at the same level of biological plausibility. PPARγ has attracted the most attention. It is closely tied to lipogenic differentiation and metabolic flexibility. In fibroblast lineages, reduced PPARγ activity is linked to myofibroblast activation and loss of a more lipogenic stromal phenotype, so restoring this pathway may help shift fibroblasts away from a persistent profibrotic state (El Agha et al., 2017). Part of the appeal of this idea is that it goes beyond simple inhibition. It raises the possibility that fibrosis partly reflects a failure of cell identity, a view consistent with the framework advanced in Section 4. PPARα plays a different role. It is more closely linked to fatty-acid oxidation and mitochondrial lipid use, and its role in pulmonary fibrosis is less clearly defined than that of PPARγ, but the biological rationale is strong (Romero et al., 2018; Angeles-Lopez et al., 2025; El Agha et al., 2017). Other lipid-sensing nuclear pathways may also matter, including liver X receptors (LXRs) and sterol-responsive programs, especially in cells exposed to altered cholesterol trafficking or oxidized lipids, including macrophages (Romero et al., 2018; Houten and Wanders, 2010; Geng et al., 2022). The picture therefore extends beyond fatty acids alone. It also includes cholesterol sensing and transcriptional responses to metabolic stress.

Across this tier, metabolic plausibility should not be confused with clinical proof. Metformin, ezetimibe, and PPAR-based strategies share a strong biological rationale and a weak translational base. They are best read as candidates for stratified trials in selected patient subgroups rather than as broadly applicable therapies.

### 6.3 Mechanism-driven, preclinical strategies: mitochondrial-redox network and ferroptosis

A third group of strategies acts further upstream, at the level of mitochondrial function, energy sensing, redox balance, and the iron-dependent phospholipid peroxidation programs introduced in Section 5. These approaches are included here not because they form a unified drug class but because they engage lipid handling and ferroptotic susceptibility from a shared mechanistic direction.

Mitochondria sit close to the center of this problem. They support fatty-acid oxidation, ATP production, and redox control. In fibrotic lungs, mitochondrial function is often impaired, which can reduce metabolic flexibility, increase oxidative stress, and make cells more likely to adopt pathogenic states (Rodríguez et al., 2025; Liang et al., 2023; Tong et al., 2024). Energy-sensing pathways are tightly coupled to this. AMPK, mTOR, and hypoxia-related signaling shape how cells respond to stress, and in fibrosis these pathways can become locked into maladaptive patterns. Epithelial cells repair less effectively, fibroblasts maintain anabolic programs, and immune cells remain activated. Rebalancing these pathways may help shift the tissue environment in a less pathogenic direction (Rodríguez et al., 2025; Liang et al., 2023; El Agha et al., 2017). Redox-directed approaches matter for the same reason. Oxidative stress lies at the junction of lipid dysregulation and ferroptotic susceptibility, and interventions that reduce reactive oxygen species or strengthen endogenous antioxidant systems may loosen the cycle linking lipid peroxidation to inflammation and matrix deposition (Wang C. et al., 2023; Nishizawa et al., 2023). Fibrosis is unlikely to yield to a single metabolic lever. Mitochondrial dysfunction, altered energy sensing, and redox imbalance are best understood as parts of a broader lipid-redox network rather than as isolated targets.

Ferroptosis has emerged as a particularly coherent target within this network. It lies at the intersection of lipid dysregulation, iron imbalance, and antioxidant failure, and three broad strategies have been pursued. One aims to reduce iron-dependent oxidative pressure. A second seeks to limit phospholipid peroxidation more directly. A third aims to strengthen endogenous defense systems, including glutathione-dependent pathways (Wang C. et al., 2023). The supporting evidence is mainly preclinical. In bleomycin-induced lung fibrosis, reducing ferroptosis or iron accumulation lessens fibrotic change, which establishes the pathway as modifiable and biologically relevant even though human therapeutic validation is still lacking (Pei et al., 2022). The appeal of this strategy goes beyond simple cytoprotection. Limiting ferroptosis may reduce the generation of oxidized lipid species, lessen epithelial injury, and weaken the signals that sustain macrophage and fibroblast engagement, as outlined in Section4.3. Caution is still needed. Ferroptosis is tightly coupled to normal redox control and lipid metabolism, so broad suppression may not always be beneficial. Its role likely changes across cell types and disease stages. At present, the strongest evidence supports epithelial protection; how best to target stromal and immune compartments is less clear (Sun et al., 2025; Wang C. et al., 2023).

Natural products and other adjunctive interventions sit at the exploratory edge of this tier. In experimental systems, some compounds reduce lipid peroxidation, improve redox balance, or dampen profibrotic signaling, and they are often considered alongside dietary or nutrient-based approaches that may influence systemic lipid balance and redox tone (Xu et al., 2020; Shaker et al., 2026; Mei et al., 2025). At this stage these findings are better treated as hypothesis-generating than practice-changing. The available data remain limited and heterogeneous, and direct antifibrotic relevance is still uncertain. Their value is largely conceptual: they widen the mechanistic frame and occasionally hint at combination strategies, but they do not yet form a mature therapeutic category.

Taken together, this tier is mechanistically attractive but clinically unproven. Its therapeutic value will depend on whether interventions can be matched to the compartments and stages where ferroptosis and mitochondrial-redox failure are most consequential, rather than applied as broad-spectrum metabolic protectors.

## 7 Conclusions and future perspectives

This review has advanced three propositions. First, lipid metabolic remodeling in pulmonary fibrosis is compartment-specific and stage-dependent, with the clearest correlative human observations in the alveolar epithelium and progressively weaker support in stromal and immune compartments. Second, phospholipid peroxidation and ferroptosis act as a downstream amplifier of these compartment-specific failures, converting metabolic stress into membrane damage, epithelial loss, and feedback into the profibrotic niche, rather than serving as an independent disease driver. Third, therapeutic strategies that engage this biology are best organized by their proximity to lipid metabolism and by the strength of their clinical support, ranging from indirectly relevant clinical anchors to mechanism-driven preclinical interventions.

Read together, these propositions point to a single conceptual shift. Lipid metabolism in pulmonary fibrosis is most usefully understood as a determinant of cell-state stability rather than as an energetic substrate of disease. Fibrotic progression can then be read as the failure of multiple lipid-supported cell-state programs: a destabilized regenerative AT2 state, a locked-in myofibroblast identity, and a sustained monocyte-derived profibrotic macrophage niche. This framing brings the otherwise disparate observations of Sections 3 and 4 under a common explanatory frame and clarifies why no single metabolic intervention is likely to address the disease in full.

It also exposes the gap between current therapy and the biology it ought to engage. Existing antifibrotic drugs and PDE4B inhibition slow progression and have clinical support, but they do not directly correct the divergent metabolic abnormalities seen across cell populations. The clearest translational signal still comes from epithelial lipid dysregulation, where ferroptosis-oriented and lipid-restorative strategies have the most coherent rationale. Stromal and macrophage programs remain biologically plausible but less clearly actionable, and the field will need to distinguish causal drivers from secondary adaptation more convincingly before targeting them with confidence.

The next challenge is therefore precision rather than scope. The same metabolic pathway does not carry the same meaning in epithelial, stromal, and immune compartments, and its role can shift with disease stage. This places real demands on biomarker development and trial design. Candidate readouts include surfactant-related phospholipid composition in bronchoalveolar lavage, plasma oxylipins and ferroptosis-related lipid mediators, and iron-handling markers such as ferritin and transferrin saturation. Whether any of these can stratify patients with progressive fibrotic ILD by their likely response to a lipid-targeted or ferroptosis-targeted intervention is still an open empirical question, but it is one that the next-generation of trials should aim to answer rather than postpone.

Three research priorities follow from this view. The first is to define compartment- and stage-resolved metabolic heterogeneity in human disease, drawing on single-cell, spatial, and lipidomic platforms rather than bulk tissue averages. As illustrated by single-cell profiling of human IPF alveolar epithelium, bulk tissue measurements fail to capture stage-specific fatty acid oxidation defects restricted to injured AT2 subpopulations (Angeles-Lopez et al., 2025). The second is mechanistic dissection. Studies need to separate metabolic changes that drive cell-state failure from those that follow it, ideally using compartment-specific genetic or pharmacological perturbation in models that recapitulate human disease features. Lineage-tracing studies focused on macrophage ontogeny serve as a representative example: this compartment-restricted genetic manipulation clearly distinguishes cell-intrinsic metabolic programs that initiate fibrotic injury from metabolic shifts arising as secondary consequences of tissue scarring (Misharin et al., 2017). The third is therapeutic matching. Interventions, whether repurposed metabolic agents, PPAR-related modulators, or ferroptosis inhibitors, need to be tested in the compartments and stages where their target biology is most active, and combined with established antifibrotic therapy where mechanistic complementarity is plausible.

The pressing question is no longer whether lipid metabolism is involved in pulmonary fibrosis. The more useful question is which abnormalities are actionable, in which cells they matter most, and when intervention is most likely to change disease course.

While this review systematically integrates compartmental lipid metabolism disorders and ferroptosis-mediated fibrotic amplification, several limitations deserve consideration, together with the mitigating approaches we adopted and suggestions for future research. Our literature search only included English peer-reviewed articles without grey or non-English publications, which may introduce publication and language bias; we offset this deficiency by incorporating two high-quality phase III clinical trials from top respiratory journals, and subsequent reviews should expand multilingual databases and grey literature retrieval to minimize such bias. Most mechanistic conclusions are derived from acute mouse fibrosis models, creating uncertainty when extrapolating results to chronic human pulmonary fibrosis, and we have adopted cautious interpretation for all preclinical animal-derived mechanistic findings to avoid overextrapolation to human disease. Besides, lipid- and ferroptosis-targeted interventions lack sufficient human clinical validation, which we objectively reflected via tiered classification of therapies by clinical evidence maturity in Table 2; further biomarker-stratified clinical trials and verification using human lung organoids are required to advance translational research.
